# Eliciting Care Management Preferences with Engaged Caregivers and Individuals With Mild Cognitive Impairment

**DOI:** 10.1093/geroni/igaa057.2586

**Published:** 2020-12-16

**Authors:** Tabassum Majid

**Affiliations:** University of Maryland, Baltimore County, Baltimore, United States

## Abstract

The goal of this study was to engage individuals with mild cognitive impairment (MCI) in developing a survey instrument that assists in the selection and prioritization of meaningful treatment outcomes in health care decision making. A multi-stakeholder advisory panel was engaged throughout the first qualitative phase of the study. 15 in-depth, guided interviews were conducted with caregiver-care recipient dyads who had been recently diagnosed with MCI. In addition to questions about meaningful outcomes, individuals were asked about quality of life and the severity of their symptoms using the Functional Attainment Staging Tool. 14 thematic concepts identified in an earlier, non-engagement-based caregiver study were all endorsed as relevant to the dyads interviewed. However, participants included 6 more thematic concepts, many of which were focused on social and community factors relevant to health care decision making. Engaging patients and caregivers allowed for focused dialogue within the interviews on shared treatment goals.

